# Giant left diaphragmatic hernia

**DOI:** 10.1093/omcr/omab023

**Published:** 2021-05-24

**Authors:** F J Voskens, R van Hillegersberg

**Affiliations:** Department of Surgery, University Medical Center Utrecht, 3584 CX Utrecht, The Netherlands

**Figure 1:**
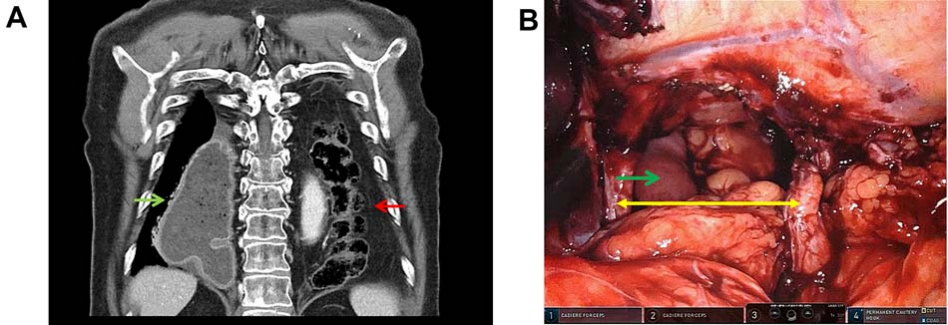
(A) Coronal image from the computed tomography demonstrating herniated colon into the left chest (red arrow) with compression on the dilated gastric conduit (green arrow). (B) Surgeon’s robotic console display with the endoscopic surgical view of the diaphragmatic hernia (yellow arrows) and the gastric conduit (green arrow), after reduction of the herniated colon.

A 67-year-old man presented with progressive shortness of breath, intermittent vomiting and constipation. On examination, he had audible bowel sounds in the left hemithorax. Computed tomography revealed a giant diaphragmatic hernia allowing herniation of the entire transverse colon into the left hemithorax (Fig. 1A). The vomiting was caused by intermittent obstruction of the gastric conduit which had been formed in 2018 during a robot-assisted minimally invasive esophagectomy for adenocarcinoma of the distal esophagus. The patient underwent robotic-assisted reduction of the colon and repair of the defect using non-absorbable sutures and mesh cruroplasty (Fig. 1B). The post-operative course was uneventful and no signs of hernia recurrence were identified at follow-up. The patient reported significant improvements in breathing, exercise tolerance and bowel habits. The incidence of hiatus hernia following esophagectomy is between 7 and 10% [1]. It is however expected to occur more frequently as a result of les scar formation following minimal invasive surgery [2]. Surgical repair is indicated in patients with symptomatic bowel obstruction and/or respiratory symptoms [3]. Robotic systems are increasingly used in redo surgery due to demanding adhesions and changed anatomy [4]. The 3D vision and wristed instruments of the robot offer benefits in delicate dissections and suturing in confined spaces.
